# Proper Read Filtering Method to Adequately Analyze Whole-Transcriptome Sequencing and RNA Based Immune Repertoire Sequencing Data for Tumor Milieu Research

**DOI:** 10.3390/cancers12123693

**Published:** 2020-12-09

**Authors:** Sungyoung Lee, Seulki Song, Sung-Soo Yoon, Youngil Koh, Hongseok Yun

**Affiliations:** 1 Center for Precision Medicine, Seoul National University Hospital, Seoul 03082, Korea; sy@snuh.org; 2 Cancer Research Institute, Seoul National University College of Medicine, Seoul 03080, Korea; sosug32@snu.ac.kr (S.S.); ssysmc@snu.ac.kr (S.-S.Y.); 3 Department of Internal Medicine, Seoul National University Hospital, Seoul 03080, Korea

**Keywords:** T-cell receptor, immune repertoire, immune repertoire sequencing, whole transcriptome sequencing, clonotype

## Abstract

**Simple Summary:**

The recent advancement in high-throughput sequencing has become indispensable for immune-genomics and profiling the T- and B-cell receptor repertoires. Immune repertoire sequencing (IR-seq) and whole transcriptome sequencing (WTS) can be implemented to investigate and quantitatively characterize the complex pattern of the CDR3 region. We conducted T-cell diversity analysis result comparisons of these sequencing methods and suggest an intuitive approach to discriminate reliable TCR sequences and clonotype patterns from capturing errors. Although bulk-RNA sequencing is commonly used for cancer analysis, we confirmed capturing highly enriched TCR transcripts with IR-seq is more reliable for accurate immune repertoire discovery, and singleton read filtering criteria should be applied to capture true clonotypes from error-prone sequencing data. The use of such well-established data and analytical methodologies can broaden understanding of antigen specificity in immunity and enabling efficient therapeutic antibody finding.

**Abstract:**

Analysis of the T-cell receptor (TCR) repertoire is essential to characterize the extensive collections of T-cell populations with recognizing antigens in cancer research, and whole transcriptome sequencing (WTS) and immune repertoire sequencing (IR-seq) are commonly used for this measure. To date, no standard read filtering method for IR measurement has been presented. We assessed the diversity of the TCR repertoire results from the paired WTS and IR-seq data of 31 multiple myeloma (MM) patients. To invent an adequate read filtering strategy for IR analysis, we conducted comparisons with WTS results. First, our analyses for determining an optimal threshold for selecting clonotypes showed that the clonotypes supported by a single read largely affected the shared clonotypes and manifested distinct patterns of mapping qualities, unlike clonotypes with multiple reads. Second, although IR-seq could reflect a wider TCR region with a higher capture rate than WTS, an adequate comparison with the removal of unwanted bias from potential sequencing errors was possible only after applying our read filtering strategy. As a result, we suggest that TCR repertoire analysis be carried out through IR-seq to produce reliable and accurate results, along with the removal of single-read clonotypes, to conduct immune research in cancer using high-throughput sequencing.

## 1. Introduction

The most important function of the immune system is to recognize antigens, defend by increasing the number of immune cells that make antibodies and kill external pathogens such as bacteria and viruses [1]. In particular, the function of the adaptive immune system is important because disease occurs when our immune system halts the recognition of external pathogens or reacts excessively [2,3]. T-cell receptors (TCRs), which are located on the surface of T-cells and selectively recognize major histocompatibility complexes on the surface of antigen-presenting cells [4,5], accelerate T-cell responses and rapidly provoke immune responses by secreting cytokines [6]. Diversity and flexibility are essential for these TCRs to respond to extremely diverse antigens, and these are determined by random rearrangements of VDJ genes during the development of T-cells to produce a variety of complementarity-determining region (CDR) sequences for each clone [7].

To understand the complex TCR diversity, researchers have previously used Southern blot hybridization or PCR analysis [8,9], but with the rapid development of next-generation sequencing (NGS), high-throughput analysis of the immune system has been realized by implementing the analyses of T- and B-cell-specific repertoires at the sequence level, represented as clonotypes [10,11]. Here, two major NGS-based methods have been used to identify CDR sequences that determine unique clonotypes [12,13]. One is immune repertoire sequencing (IR-seq), a deep sequencing method in which gDNA or RNA is amplified to perform extensive TCR profiling to elucidate the entire immune system [11,14]. IR-seq enables the quantification of each CDR3 sequence in unprecedented depth and accuracy within any sample of interest [12,15]. The other is RNA-bulk sequencing, which is used for transcriptional landscape analysis of human genomes [16]. RNA-seq has the advantage of being able to perform a variety of analyses, including immune cell studies, without wasting additional tissue at a much lower price than IR-seq [17,18]. Owing to the cost performance, many researchers have conducted RNA-seq to perform both transcriptomic and IR analyses at no extra cost.

There have been a number of studies that compared and grasped a similar degree of TCR clones extracted from RNA-seq and IR-seq [13,19,20]. However, those studies were not conducted to determine whether it is informative enough to selectively extract the actual CDR3 sequence among all transcriptome sequences to identify the potential repertoires. According to previous literature, RNA-seq-based TCR profiling is limited in detecting a wide range of TCR regions since it infers the results by extracting only CDR3 regions belonging to a portion of the entire transcript [20]. Moreover, due to their low abundance, capturing the mostly highly enriched TCR transcripts could be a more reliable but incomplete estimation of TCR repertoires from other random reads [13,20]. However, no appropriate quality control approach for IR-seq has been proposed, which limits the credibility of the immune repertoire estimated from extensively complex IR-seq reads.

In this study, we conducted a systematic comparison of T-cell diversity analysis results to determine how accurately paired RNA-seq and IR-seq describe the immune repertoire and specify the absence of a systematic impediment associated with capturing the complex pattern of the CDR3 region. For the analysis, specimens from multiple myeloma (MM) patients with abnormally differentiated or proliferated plasma cells, leukocytes responsible for the immune system in bone marrow (BM), were used. Following classification with the CD138 marker, paired RNA-seq and IR-seq were performed on each sample to examine the characteristics of the immune system of MM patients at the patient level. In particular, we present an intuitive approach for reducing putative errors associated with IR-seq to increase the accuracy of the estimated immune repertoire. This led us to assess T-cell clonality and the results of both sequencing methods and quality controls.

## 2. Results

### 2.1. Study Population and Basic Characteristics of TCR Sequences

**Figure 1:**
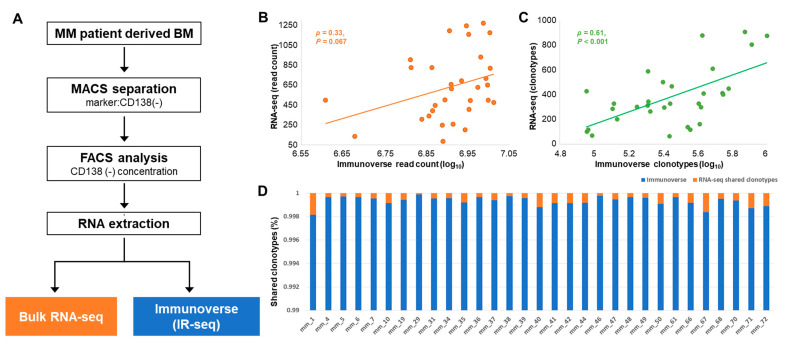
T-cell repertoire distributions in MM patients. (**A**) Study scheme of the procedure applied for TCR repertoire analysis. Bone marrow from MM patients was subjected to MACS with the CD138 marker, and RNAs from CD138^−^ cells were extracted for whole-transcriptome and TCR repertoire sequencing. (**B**) Distribution of total read counts and (**C**) the number of total clonotypes detected from each sample from both bulk RNA-seq and IR-seq are shown. (**D**) The percentage of shared TCR amino acid clonotypes. The total TCR repertoire from IR-seq is represented in blue bars, while the percentage of shared clonotypes, which were also detected from bulk RNA-seq, is represented in orange bars.

From the immune milieu portion of BM samples from 31 patients with newly diagnosed MM, we performed both IR-seq using Immunoverse [21] from ArcherDX (Boulder, CO, USA) and RNA-seq on each sample (Figure 1A). Here, only immune cells from the BM of the patients were isolated and analyzed through classification with the myeloma marker CD138. The clinical characteristics of the MM patients are listed in Table 1. Of 31 newly diagnosed MM patients, 17 were males and 14 were females. The mean age of the total cohort was 65.9 years (range 47–80), and the average CD138 negative rate was 0.75 after magnetic-activated cell sorting. Eight (25.8%) patients were classified as R-ISS I based on the revised international staging system for myeloma; 16 (51.6%) as R-ISS II; and 7 (22.6%) as R-ISS III.

The sequencing characteristics (e.g., total mapped reads to human reference genome with bulk RNA-seq using STAR alignment, TCR region mapped reads and number of unique TCR clonotypes) shown in Table 2 suggest that the coverage depth of each CDR3 was captured at high rates with IR-seq, which agrees with our expectation, and more diversified amino acid (AA) clonotypes were detected than those detected with RNA-seq. While the numbers of TCR region mapped reads from IR-seq ranged from 4,050,594 to 10,274,849, those from RNA-seq ranged from only 85 to 1273 of 222,816 to 230,246 total reads. Similarly, the number of unique clonotypes from IR-seq was 9,932,683, which was exceedingly higher than that from RNA-seq (11,616 unique clonotypes). Few clonotypes derived from the same AA sequences spanned multiple different nucleotide TCR sequences within a donor, with the majority of clonotypes belonging to a single time point in both sequencing types but more frequently in IR-seq. Based on the previous findings that IR-seq provides an accurate measure of TCR diversity [22] and the results from our data, we assumed the TCR sequencing results as a set of true signals and compared them with the repertoire of RNA-seq results in each sample.

### 2.2. TCR Repertoire Diversity in MM Samples from IR-seq and RNA-seq

To identify the diversity of the TCR repertoire accurately, we first substantiated the CDR3 AA sequences by mapping all reads to the TCR antibody references and the proportion of TCR genes for each sequencing method.

First, the comparison suggested that RNA-seq showed a wide variety of both read counts and clonotypes among samples, caused by sparsity, while IR-seq showed stable distributions (Figure 1B,C). The TCR repertoire results obtained from RNA-seq are not only outspread in the range of minimum and maximum values but also disparate within all samples. Previous reports [19] have shown that extracting IRs from RNA-seq depends greatly on the length of the reads whether they are single- or double-ended and only large clonotypes containing a significant number of T-cells can be quantified, which explains our results extracted from RNA-seq are unstable per sample.

Second, we identified some patterns that suggested that RNA-seq was less efficient than IR-seq. Although the total number of mapped reads from bulk RNA-seq data using STAR alignment did not show a very large difference among samples (Figure S1), the distribution of reads mapped to the TCR region from RNA-seq within all samples was atypical. In addition, the unique clonotypes extracted from RNA-seq data were less than 0.001% of the total amount extracted from IR-seq data (Figure 1D). In contrast to IR-seq, which sequences with efficient TCR library targets, RNA-seq can only read a small portion using fragments randomly scattered throughout the whole transcriptome. Therefore, assuming the characteristics and specific landscape of IRs using bulk RNA-seq data is not as efficient as IR-seq, and there are various limitations.

Third, an investigation of TCR genes showed substantial differences between the sequencing methods. The variable gene (V gene) usage of TCR in each patient, which was measured by relative abundance against the richest gene’s abundance, is depicted in Figure S2A and the most abundant TRBV genes were represented in Figure S2B to emphasize its differences between IR-seq and RNA-seq. There were 16 statistically significant (Welch’s *t*-test *p* < 0.05) differences between the two sequencing methods among all 50 V genes. Most of the genes (TRBV5-1, TRBV7-9, TRBV7-2, TRBV6-1, TRBV10-3, TRBV7-3, TRBV18, TRBV15, TRBV10-1, TRBV11-3, TRBV11-1, TRBV4-2 and TRBV4-3), except for three (TRBV24-1, TRBV25-1 and TRBV5-6), were highly expressed in IR-seq, but it was challenging to make a clear distinction because of sample variation. The reason for this observation is that the distribution of samples containing each V gene varied widely, and the difference among each sample was even more pronounced with RNA-seq. Likewise, those of joining (J) genes manifested similar patterns, and sample variation was more prominent in the RNA-seq results than in the IR-seq results (Figure S2C). This result indicates that sample variation may indeed be due to clinical differences, as the immune status is diversified depending on the patient group. However, the low resolution of RNA-seq reads more likely represents the hassle associated with identifying the complex diversity of the TCR region, which was successfully drawn from IR-seq. Therefore, we analyzed the total number of mapped reads that confidently verified each clonotype.

### 2.3. Proper Read Filtering and Clonotype Abundances at the Single-Patient Level

**Figure 2:**
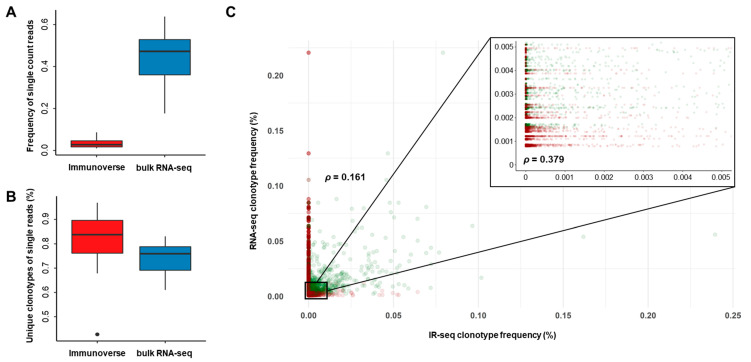
Distribution of single reads coding new amino acid clonotypes in TCR repertoires. (**A**) The frequency of single reads from each sequencing method. (**B**) The percentage of unique clonotypes referenced by single reads from each sequencing method. (**C**) Correlation of the shared TCR clonotype frequencies generated by IR-seq and bulk RNA-seq. Each dot represents each sample sharing the same amino acid clonotypes. Frequencies of removed TCR clonotypes with single reads are represented with red circles. Zoomed-in scale where the most clonotypes with single reads existed (from 0 to 0.005).

With both sequencing methods, the read count distribution of TCR sequences pinpointed that the majority of unique clonotypes was characterized by a single read. From IR-seq, clonotypes with a single read were almost 3.5% of the total read count, and they denoted 81.9% of the clonotypes. Likewise, RNA-seq denoted 74.6% of all clonotypes with a singleton, but their proportion in view of read counts was 44.6%, which was substantially larger than that denoted by IR-seq (Figure 2A,B). These results suggest that clonotypes with single read that are virtually impossible to distinguish from mapping biases or sequencing errors represent a large proportion of the total clonotypes and that they should be removed if they cannot be reliably proved. However, there are still no recommended values that must be carried out to find a clear IR pattern in the current IR analysis methods.

To determine an optimal threshold for selecting reliable clonotypes, we first reviewed the distribution of the read counts for clonotypes in our 31 samples and selected the filtering criteria based on these results. In total, 37,668 clonotypes were found in at least two samples in all 31 IR-seq results (Figure S3A). However, removing singleton reads reduced the number of shared clonotypes to 23,905, approximately 37% of the total. When clonotypes with less than two read counts and less than three read counts were removed, 21,959 and 20,857 common clonotypes remained, respectively, which was not much different than when clonotypes from a single read were removed. The shared clonotype (i.e., the clonotype with overlapping IR-seq and RNA-seq results from matched samples) was also compared, and it was markedly reduced by read count filtering. The removal of one count read on both sides eliminated approximately 63.7% of the shared clonotypes, while the removal of doublets and triplets eliminated up to 82.7% and 88.7%, respectively (Figure S3B). Despite the reduction in shared clonotypes, it is inevitable to draw the conclusion that clonotypes with insufficient reads cannot be distinguished from putative erroneous reads, since we performed strict read validation before mapping, as described in the Materials and Methods section.

**Figure 3:**
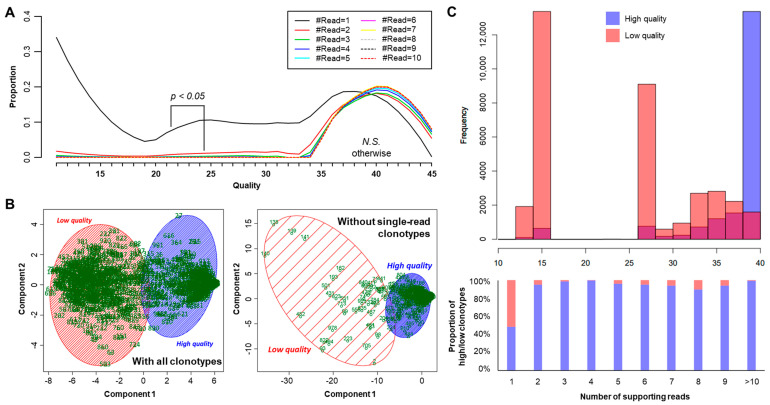
Comparison of mapping quality and effect of low-quality clonotype filtering. (**A**) Group-wise comparison of mapping quality score distribution by the number of supporting reads. Only one *p*-value of Kolmogorov–Smirnov test is depicted between the distributions of quality scores from # reads = 1 vs. 2, while the others are summarized as a text below the legend (*N.S.*, Not Significant) (**B**) Results of PCA and *k*-nearest neighbor clustering before (left) and after (right) single-read clonotype filtering. (**C**) Distribution of quality scores between low- and high-quality clonotypes (top) and their group-wise proportion by the number of supporting reads (bottom).

Second, we compared the mapping qualities of both single-read and other clonotypes to identify whether single-read clonotypes harbor quality issues. In this respect, all clonotypes were grouped by their supporting read numbers, and the group-wise distributions of quality scores were compared (Figure 3A). As a result, we found that only the clonotypes with single support showed statistically significant differences (adjusted Kolmogorov–Smirnov test *p* < 0.05, Figure 3A) with other groups of clonotypes, which also supports our suggestion of filtering single-read clonotypes. Moreover, a further clonotype-level analysis in which principal component analysis (PCA) was used showed that the removal of single-read clonotypes dramatically improved clonotype quality, since the majority of low-quality clonotypes were supported by a single read (Figure 3).

Based on these results, we removed all clonotypes annotated with a single count and compared their distribution once more. Consequently, 31–746 read counts targeting 2840 unique clonotypes and 3,987,468–10,072,564 reads covering 1,654,551 unique clonotypes were identified from RNA- and IR-seq, respectively. The distribution of the shared clonotypes showed substantial differences that changed with read filtration. As indicated as red circles in Figure 2C, clonotypes with very low ratios from both sequencing methods as well as those with the highest proportions on either side were removed after the removal of clonotypes with a single read. In addition, the overall number of correlations increased, confirming that the results are more accurate when analyzing and comparing IRs. The correlation coefficient values of the two methods also increased significantly after removing single-read clonotypes (Figure 2C).

### 2.4. Assessment of TCR Genes from IR-seq and RNA-seq after Read Filtering

**Figure 4:**
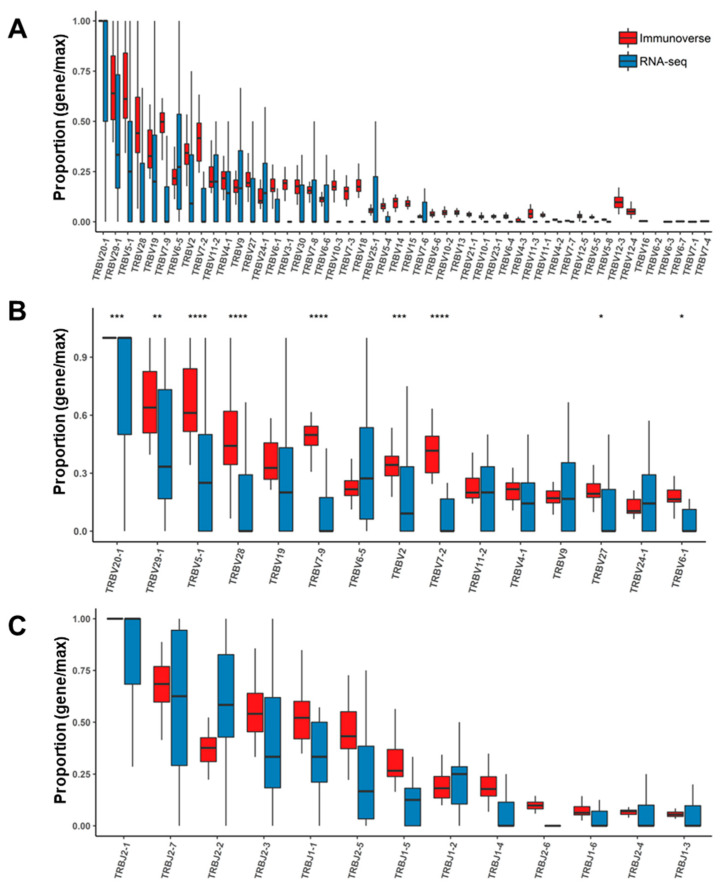
Comparisons of TCRB gene usage after single read removal. (**A**) TRBV genes with usage differences between two sequencing methods. The y-axis depicts the proportion of each gene among all TRBV genes from each sample (p proportion of each gene/maximum gene proportion). The value for the most frequent gene was equal to one. The x-axis is sorted by the highest proportion among all genes. Distribution and comparison of the most abundant (**B**) TRBV genes and (**C**) all TRBJ genes. * *p*  ≤  0.05, ** *p* ≤ 0.01, *** *p* ≤ 0.001, **** *p* ≤ 0.0001, *t*-test.

Upon reconfirming the pattern of all TCR genes after read filtering, the characteristic elements appearing in the whole samples were well represented, and the difference in the results between the two methods was clearly denoted, as shown in Figure 4 that depicts the sample-wise relative abundances of TCR genes against the most expressed gene in each category. The importance of the expression level and overall proportion of most TCR genes could be confirmed by IR-seq, but there were several genes whose RNA-seq results could not be determined (Figure 4A, genes TRBV12-3 to TRBV7-4). With our observation that most genes from RNA-seq vary substantially among samples, we believe that RNA-seq has a weaker performance in identifying patterns in TCR genes than IR-seq (Figure 4). This is believed to be due to the low TCR gene extraction from RNA-seq, which suggests that RNA-seq results alone may indicate a misunderstanding of T-cell diversity in certain patients or genes.

**Figure 5:**
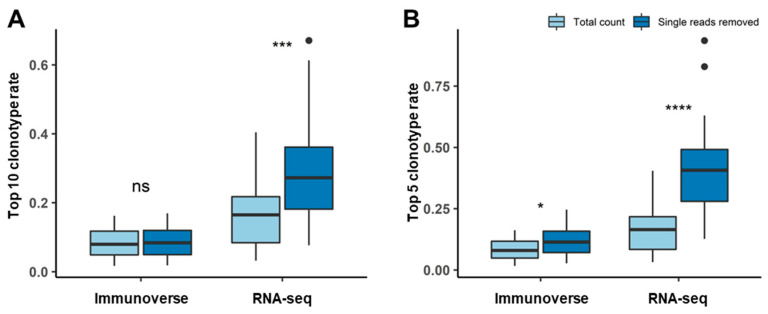
The most frequent clonotype rate changes with outliers represented as dots. Percentage of the most frequent TCR CDR3 AA sequences before and after single read removal from both sequencing methods. There were no significant differences in the (**A**) top 10 or (**B**) top 5 most frequent AA sequences with IR-seq, but the ratio of the top clonotypes increased after the singleton reads were removed in RNA-seq. (ns, not significant; * *p* < 0.05, *** *p* < 0.001; **** *p* < 0.0001).

Finally, we evaluated to what extent our single read filtering affects the clonotype measurement. Here, the clonal expansion of the TCR repertoire was further assessed by calculating the frequency distribution of the CDR3 AA sequences. After removing singleton reads, RNA-seq was found to yield fewer than 20 clonotypes in two samples. Comparison of the ratio of the top 10 clonotypes before and after clearing singleton reads in both sequencing methods revealed that the proportion of total AA sequences was not significantly different (Welch’s *t*-test, *p* = 0.82) despite the large amount of clonotype removal in IR-seq (Figure 5A). However, the ratio of the top 10 clonotypes was slightly (2% on average) increased after the singleton reads were removed in RNA-seq. In addition, comparison of the proportions of the top five clonotypes yielded the same results as the top 10 (Figure 5B). This result indicates that unstable results from RNA-seq can mislead the interpretation of clonal expansion, and such trends are largely dependent on how the analysis pipeline is set, such as alignments and filtering criteria.

### 2.5. Repertoire Inference Using Random Sampling

**Figure 6:**
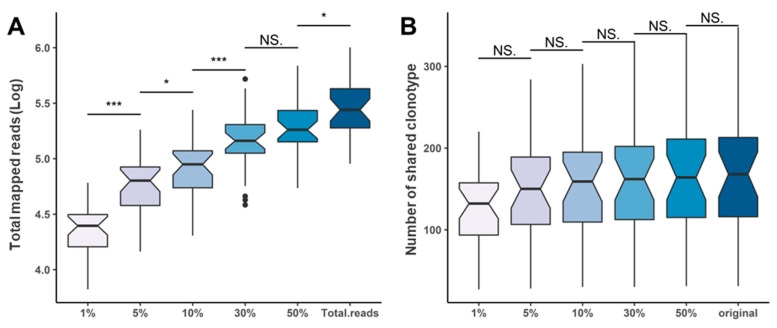
TCR clonality pattern in randomly downsampled TCR reads with outliers represented as dots. (**A**) Changes in sequencing read volume from 1% to 50% for downsampling analysis. (**B**) Distribution of shared clonotypes detected with both downsampled TCR throughput sequencing and raw bulk RNA sequencing. No significant differences in shared clonotypes or the number of clonotypes identified simultaneously in the RNA-seq results were found. (NS., not significant; * *p* < 0.05; *** *p* < 0.001).

To overcome the limited repertoire approach with scant evidence for RNA-seq, RNA-seq with extremely high-depth or additional primers to cover the whole TCR region in detail can be used under the rationale of an optimal sequencing depth. In this respect, we conducted a simple simulation to determine how the sequencing depth affects the IR results by adjusting the total sequencing depth using randomized sampling. We compared the number of unique clonotypes shared with the RNA-seq results, decreasing the read depth sequentially by 1–60% of the total Immunoverse sequencing reads. The total number of unique clonotypes found in all samples increased proportionally with the cumulative sequencing depth because of random sampling (Figure 6A). Even so, no significant differences in sequencing depth were found for the number of clonotypes identified simultaneously with RNA-seq results (Figure 6B). From our observations, even if the change in Immunoverse sequencing depth was reduced by 1%, the proportion of shared clonotypes was almost the same, and there was no significant effect on extracting the true clonotypes from each sample.

## 3. Discussion

The potential to determine precise TCR sequences is critical for understanding the specificity and flexibility of the T-cell environment [10,23]. However, due to complex TCR sequences, massive amounts of sequencing data are required to reduce bias and extract faithful results [11,24]. Many consequences have been derived using various sequencing methods to characterize the region of the TCR transcript, but, in practice, it is necessary to determine whether these methods yield accurate and precise results.

In this respect, our study provides not only reliable and systematic comparison of TCR repertoires’ detection using 31 MM samples with two different sequencing methods used to characterize immune cells, but also an intuitive and effective filtering approach to measure credible immune repertoire. While IR-seq has been regarded the most prominent method for deep scanning of TCR region with less bias [22,25], no large-scale and systematic side-by-side analysis of both IR- and RNA-seq has been conducted to demonstrate such advantages. In terms of IR analysis, the sequencing method must ensure that the analysis is performed by precisely comparing the resulting CDR3 sequences.

Our results from a large-scale dataset of 31 samples of both RNA- and IR-seq showed that IR-seq is a much more accurate reader of wider TCR sites than RNA-seq, and the greatest coverage depth of each CDR3 was captured at a high rate. From our extensive comparison, IR-seq can successfully capture the complex composition of clonotypes with substantially higher depths, thus providing much more stable and reliable results than RNA-seq, which suffers from highly variable and sparse TCR region capture.

Moreover, we demonstrated that the quality control of IR-seq requires sufficient filtering criteria to ensure robust measurement of immune repertoires, by showing our approach can efficiently eliminate potential bias from clonotypes with a single read while maintaining a major proportion of the shared clonotypes from both sequencing methods. Finally, we also confirmed smaller throughput of IR-seq is sufficient to identify most of the shared clonotypes using our random sampling approach, which can provide cost-effectiveness in certain types of immune repertoire studies.

## 4. Materials and Methods

### 4.1. Sample Collection and Processing

The 31 newly diagnosed MM patient samples investigated in this study were collected after informed consent was provided for study participation at Seoul National University Hospital. Every patient underwent BM sampling at the time of active disease [26,27]. For the isolation of CD138^−^ cells directly from whole BM samples, the mononuclear cell (MNC) layer was first depleted using gradient centrifugation with Ficoll–Hypaque solution. Separated MNCs were then released with MACS buffer followed by incubation with 20 µL of CD138 magnetically labeled microbeads, and then 10 µL of CD138-PE were added to stain CD138 cells. The CD138^−^ subset was isolated from MNCs by negative magnetic column selection using a MACS separator (Miltenyi Biotec). Afterwards, the fluorescence levels of isolated cells were determined by flow cytometry (Attune NxT, Life Technologies) using CD138 antibodies to confirm the actual concentration. Total RNA was extracted using TRIzol reagent (15596018, Thermo Fisher Scientific, Waltham, MA, USA) according to the manufacturer’s instructions. RNA concentration was measured with a NanoDrop spectrophotometer (NF-1000, Thermo Fisher Scientific, Waltham, MA, USA).

### 4.2. RNA Sequencing

The DNase step was performed before library construction to remove RNAs with DNA contamination, and RNA integrity and quantity were assessed using electrophoresis (Agilent Technologies 2100 Bioanalyzer). The transcriptome sequencing libraries were constructed with the TruSeq RNA Access Library Prep Kit (Illumina Inc., San Diego, CA, USA), and adapter-ligated fragments were then PCR amplified and gel purified. The libraries were sequenced on an Illumina HiSeq 2500 platform using paired-end run mode with a 100 bp read length following the manufacturer’s instructions.

### 4.3. Immunoverse Sequencing

Identical RNA samples after transcriptome sequencing were used for TCR library preparation. TCR libraries with molecular barcoded (MBC) adaptors to amplify the T-cell receptor alpha/delta and beta/gamma chains were constructed with 400 ng of RNA using the Immunoverse™ kit (ArcherDX Inc., Boulder, CO, USA) after PCR amplification. Sequencing of the prepared TCR library was then conducted on a HiSeq 4000 platform (Illumina Inc., San Diego, CA, USA), and 2 × 150 cycles were performed on the ArcherDX platform.

### 4.4. Data Analysis and TCR Repertoire Extraction

For IR-seq, detected TCR sequences were aligned to the human reference V, D, J and C genes [28] of TCRs using MiXCR [29]. Overlapping fragmented sequencing reads which partially aligned were assembled into CDR3-containing contigs and clonotypes and then extracted after sequencing quality score filtering and error correction [29]. TCR repertoires from bulk transcriptome sequencing data were also determined using MiXCR, which uses parameters (-p rna-seq) specifically optimized for RNA-seqas recommended by the maintainer’s webpage (https://mixcr.readthedocs.io). Reads encoding TCR region from bulk-RNA sequencing were only aligned to human TCR genes in the same manner of IR-seq. Subsequently, identified information, including the number of total clone reads, number of clonotypes (AA sequences) and statistical TCR gene diversity, was analyzed using the tcR [30] package in R statistical software.

For the statistical analyses of mapping qualities, we first grouped all clonotypes by their number of supporting reads and extracted clonotype-wise mapping qualities for each group. From the group of single-read clonotypes, we compared their quality score distribution to that of the next largest group using the Kolmogorov–Smirnov test. Next, we drew a PCA plot of the clonotypes using clonotypes-wise mapping qualities, and then we performed *k*-nearest neighbor clustering with *k* = 2 on the first two PCs to determine the low- and high-quality clonotypes. The low-quality clonotypes were further grouped by their number of supporting reads to assess how many low-quality clonotypes were present in each group.

For the visualization of TCR genes’ usage (Figure 4), we measured relative abundance of each gene. For each V, D, J category, we first identified which gene in that category contains largest clonotypes for each sample. Then, sample-wise relative abundances of a gene were derived by dividing their clonotype counts with the largest clonotype count we identified.

For the random sampling analysis, the original sequenced reads from fastq files of Immunoverse were randomly downsampled by decreasing the sequence proportion seeds using Seqtk-1.3-r106. The extracted reads were aligned and used for TCR analysis as described above. To check the raw mapping reads from RNA-seq, reads from RNA-seq were aligned to the human genome (GRCh37) using STAR aligner [31] (version 2.5.3a).

## 5. Conclusions

In summary, we believe that Immunoverse can provide more valuable and deeper insights than bulk RNA sequencing in determining the robust immune profile and characteristics of cancer patient samples. Moreover, a proper quality control strategy for IR-seq is essential to ensure the quality of the immune repertoire, and our simple and straightforward filtering strategy successfully improved the quality of the IR-seq dataset.

## Figures and Tables

**Table 1:** Clinical characteristics of patients with multiple myeloma (MM).

Clinical Characteristics	
N = 31	N (%)
**Sample N**	31
**Gender**	
Male	17 (54.8)
Female	14 (45.2)
**Median Age (Years)**	
≤65	13 (41.9)
>65	18 (58.1)
**Average CD138 (-) rate (range)**	0.75 (0.1–22.4)
**Heavy Chain Isotype**	
lgG	21 (67.7)
lgA	6 (19.4)
lgM	NA
lgD	NA
LCD	1 (3.2)
**Light Chain Isotype**	
Kappa	17 (54.8)
Lambda	11 (35.5)
**R-ISS**	
I	8 (25.8)
II	16 (51.6)
III	7 (22.6)
**Fish Results**	
p53 deletion	4 (12.9)
p16 deletion	3 (9.7)
lgH rearrangement	10 (32.3)
1q trisomy	9 (29)
RB1 deletion	9 (29)
t (14; 16)	1 (3.2)
t (4; 14)	4 (12.9)

**Table 2:** Diversity of TCR repertoires from RNA-seq (left) and IR-seq (right).

mm_#	RNA Sequencing Results				Immunoverse Results		
	STAR Mapped Reads	Total Read Counts	Total Unique Clonotypes	Singleton Reads	Total Read Counts	Total Unique Clonotypes	Singleton Reads
MM_1	54,357,923	820	431	299	4,050,594	90,068	63,126
MM_10	48,374,912	826	504	403	7,293,770	250,572	199,844
MM_19	56,698,751	819	331	250	10,073,447	275,520	252,410
MM_29	59,981,954	85	65	54	7,761,188	272,594	272,079
MM_31	51,623,766	659	331	241	8,120,860	403,438	373,784
MM_34	48,536,509	1245	809	649	8,836,571	821,579	688,017
MM_35	49,161,059	1195	883	697	8,039,194	423,108	366,790
MM_36	43,189,398	652	416	323	9,918,279	555,803	521,536
MM_37	52,095,895	827	613	510	6,500,208	487,124	425,707
MM_38	49,316,301	258	165	124	8,195,923	408,764	378,106
MM_39	48,288,693	716	412	338	9,833,228	431,722	396,254
MM_4	34,559,031	615	451	348	8,126,107	602,704	557,693
MM_40	45,488,936	499	289	198	9,963,987	127,280	105,512
MM_41	42,258,878	202	104	71	8,761,312	90,503	86,489
MM_42	45,563,654	340	120	78	7,180,034	91,962	78,633
MM_44	53,663,728	693	470	371	8,595,247	281,018	200,700
MM_46	47,093,079	408	118	72	8,958,694	360,441	348,835
MM_47	46,913,279	477	299	237	10,274,849	254,130	202,285
MM_48	45,465,437	933	403	308	9,565,796	559,857	501,017
MM_49	50,302,951	628	300	215	9,402,588	416,450	381,187
MM_5	46,521,960	248	141	97	7,732,378	347,955	345,460
MM_50	41,180,449	392	304	250	7,324,348	176,306	126,269
MM_6	52,868,514	1177	879	712	10,053,037	1,007,123	866,380
MM_61	41,841,298	136	71	54	4,765,448	96,738	91,318
MM_66	37,559,665	503	346	259	7,836,149	205,940	163,488
MM_67	41,723,673	904	329	213	6,478,122	129,689	99,656
MM_68	45,666,045	495	313	209	9,016,842	203,599	146,692
MM_7	57,602,270	1273	912	718	9,700,140	749,213	664,845
MM_70	36,073,087	447	267	199	7,430,562	210,866	154,254
MM_71	47,691,240	1158	591	412	8,943,282	204,495	141,742
MM_72	38,540,649	306	203	156	6,900,306	135,644	114,276

## Data Availability

The data presented in this study were submitted to NCBI Gene Expression Omnibus repository, with the accession ID of GSE160302. The scripts used in the analysis are available at a public code repository (https://github.com/sungyoung-lee/irseq).

## Supplementary Materials

The following are available online at https://www.mdpi.com/2072-6694/12/12/3693/s1, Figure S1. Validation of TCR gene detection with bulk RNA-seq; Figure S2. Assessment of raw mapping proportion of TCR genes from IR-seq and RNA-seq; Figure S3. Determination of the optimal threshold value for reliable clonotype detection.
