# Supplementary material for: Proper Read Filtering Method to Adequately Analyze Whole-Transcriptome Sequencing and RNA Based Immune Repertoire Sequencing Data for Tumor Milieu Research
Source: Cancers (Basel). 2020 Dec 9;12(12):3693. doi: 10.3390/cancers12123693 (PMC7763492; doi:10.3390/cancers12123693)
Supplement: Supplementary file 1 [file cancers-12-03693-s001.zip › MM_paper_supp_Cancers_v1.pptx]

## Slide 1
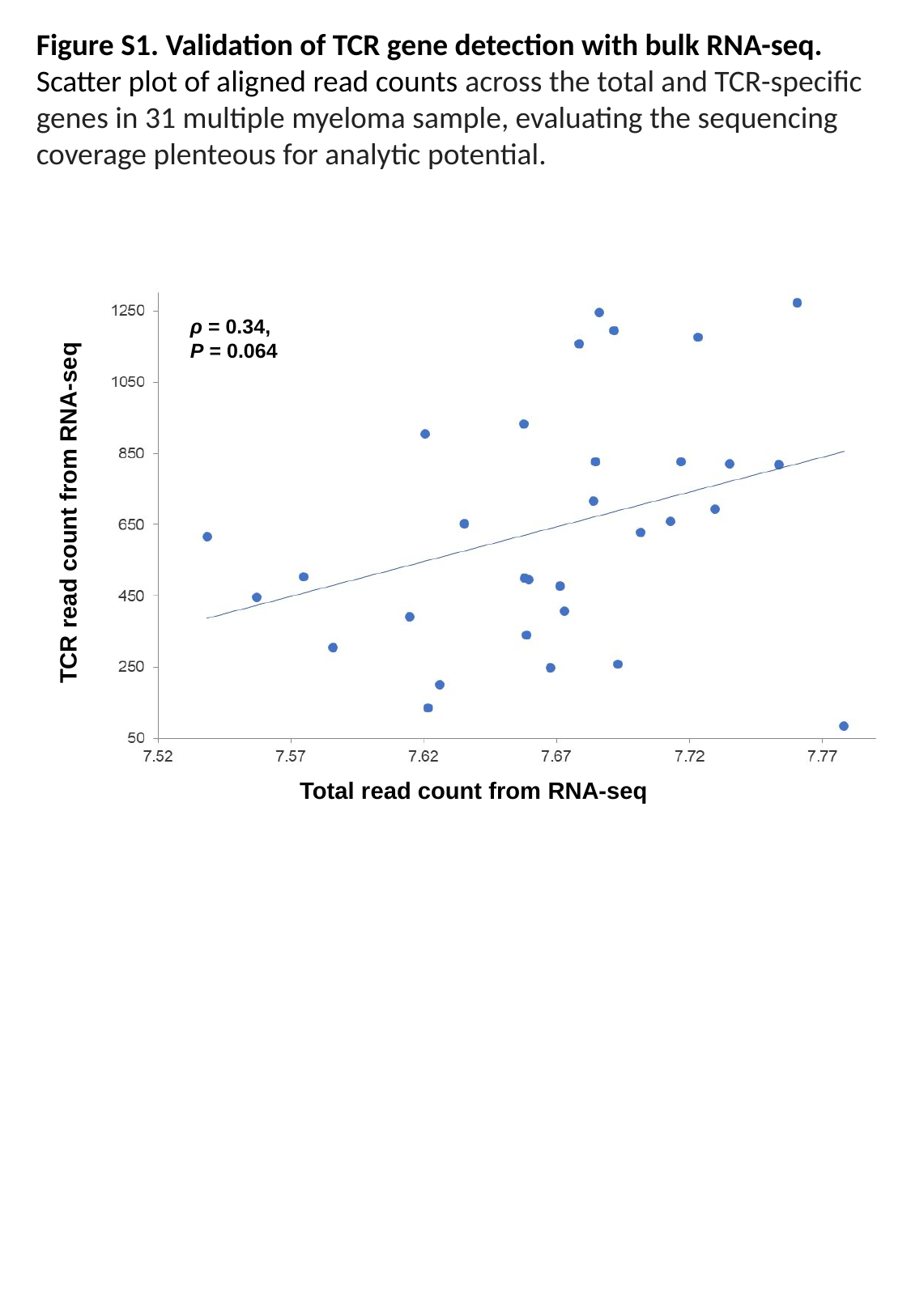

Figure S1. Validation of TCR gene detection with bulk RNA-seq.
Scatter plot of aligned read counts across the total and TCR-specific genes in 31 multiple myeloma sample, evaluating the sequencing coverage plenteous for analytic potential.
ρ = 0.34,
P = 0.064
TCR read count from RNA-seq
Total read count from RNA-seq

## Slide 2
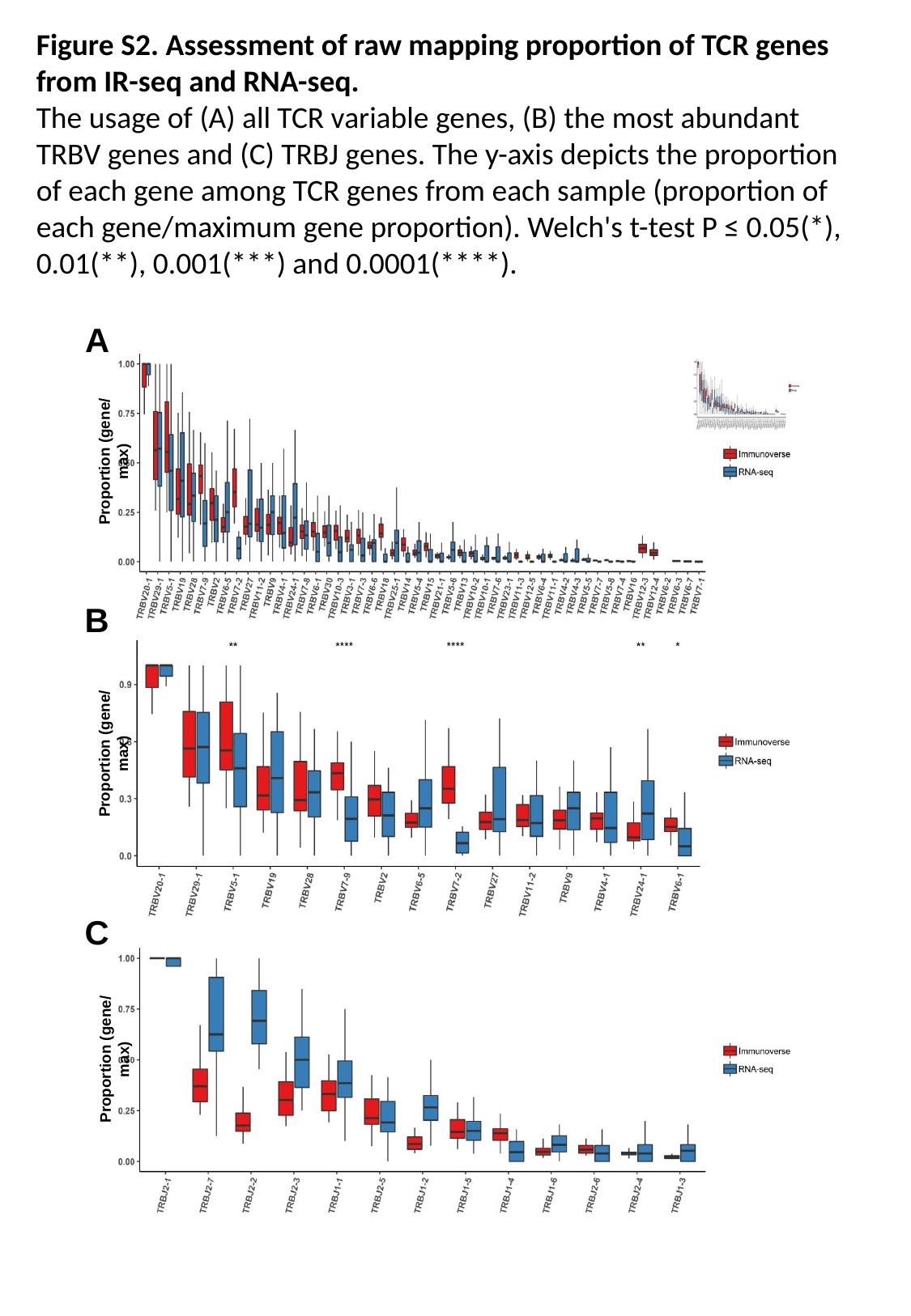

Figure S2. Assessment of raw mapping proportion of TCR genes from IR-seq and RNA-seq.
The usage of (A) all TCR variable genes, (B) the most abundant TRBV genes and (C) TRBJ genes. The y-axis depicts the proportion of each gene among TCR genes from each sample (proportion of each gene/maximum gene proportion). Welch's t-test P ≤ 0.05(*), 0.01(**), 0.001(***) and 0.0001(****).
A
Proportion (gene/max)
B
Proportion (gene/max)
C
Proportion (gene/max)

## Slide 3
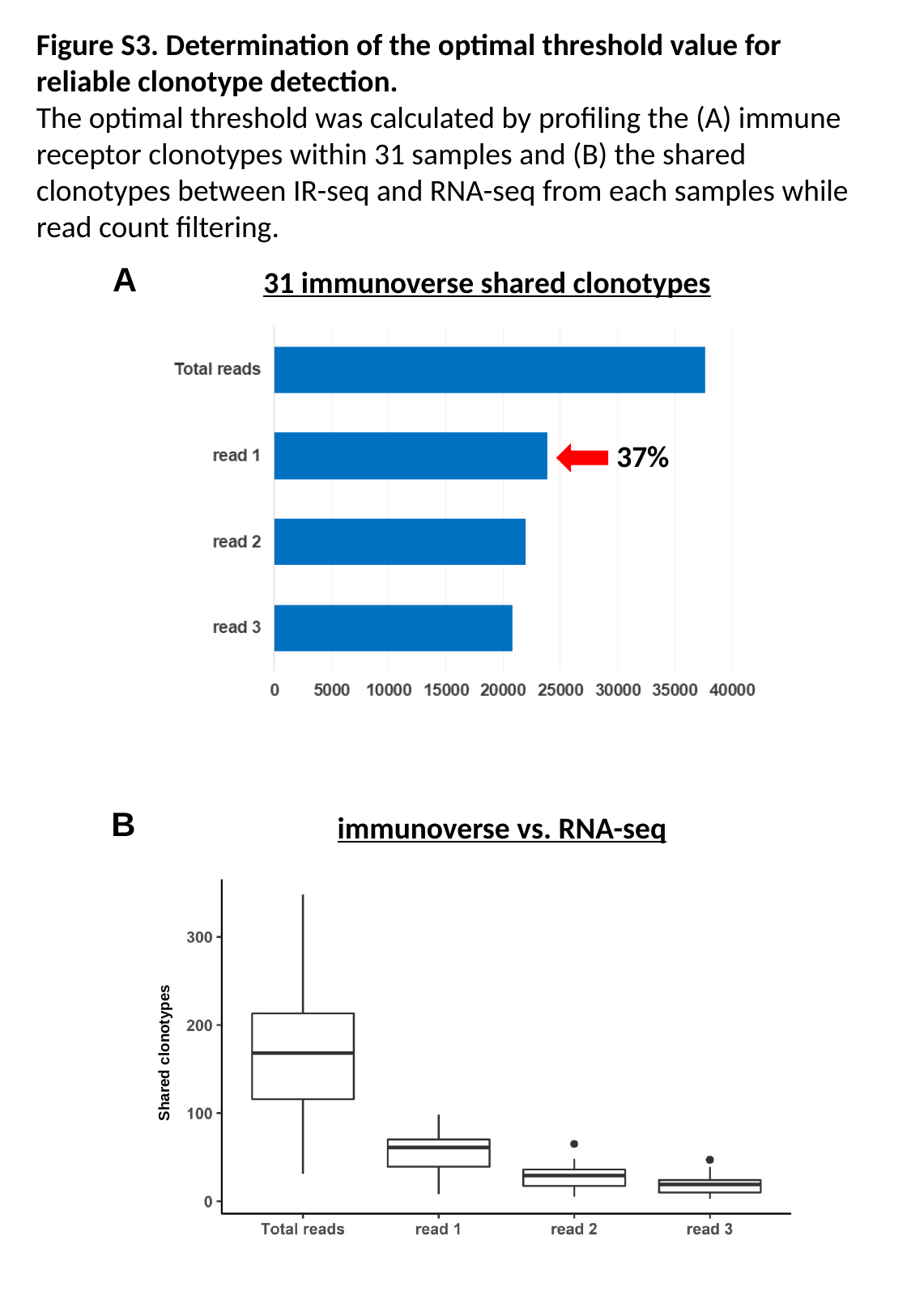

Figure S3. Determination of the optimal threshold value for reliable clonotype detection.
The optimal threshold was calculated by profiling the (A) immune receptor clonotypes within 31 samples and (B) the shared clonotypes between IR-seq and RNA-seq from each samples while read count filtering.
A
31 immunoverse shared clonotypes
37%
B
immunoverse vs. RNA-seq
Shared clonotypes
